# Neurofunctional Assessments in Lumbar Spondylosis: Outcomes After Rehabilitation Treatment

**DOI:** 10.3390/jfmk11010114

**Published:** 2026-03-09

**Authors:** Andreea Ancuta Talinga, Roxana Ramona Onofrei, Ada-Maria Codreanu, Alexandra Laura Mederle, Veronica Aurelia Romanescu, Marius-Zoltan Rezumes, Oana Suciu, Dan-Andrei Korodi, Claudia Borza

**Affiliations:** 1 Doctoral School, “Victor Babeş” University of Medicine and Pharmacy, Eftimie Murgu Sq. No. 2, 300041 Timişoara, Romania; andreea.vataman@umft.ro (A.A.T.); ada.codreanu@umft.ro (A.-M.C.); alexandra.mederle@umft.ro (A.L.M.); veronica.romanescu-pesecan@umft.ro (V.A.R.); marius.rezumes@umft.ro (M.-Z.R.); 2 Department of Rehabilitation, Physical Medicine and Rheumatology, Research Center for Assessment of Human Motion, Functionality and Disability, “Victor Babeș” University of Medicine and Pharmacy Timișoara, Eftimie Murgu Sq. No. 2, 300041 Timișoara, Romania; 3 Department of Functional Sciences—Pathophysiology, Center for Translational Research and Systems Medicine, “Victor Babeş” University of Medicine and Pharmacy, EftimieMurgu Sq. No. 2, 300041 Timişoara, Romania; borza.claudia@umft.ro; 4 Department of Medicine, Faculty of Medicine, “Vasile Goldiș” Western University of Arad, L. Rebreanu St. 86, 310048 Arad, Romania; korodi.andrei@uvvg.ro; 5 Department of Rehabilitation, Physical Medicine and Rheumatology, “Victor Babeș” University of Medicine and Pharmacy Timișoara, Eftimie Murgu Sq. No. 2, 300041 Timișoara, Romania; oanasuciu78@umft.ro

**Keywords:** lumbar spondylosis, low back pain, nerve conduction studies, electromyography, rehabilitation, exercise therapy, tibial nerve, peroneal nerve, nerve conduction velocity, muscle activation, chronic pain, conservative treatment

## Abstract

**Background**: Lumbar spondylosis is a frequent cause of chronic low back pain, often associated with radiculopathy. Although imaging evaluation is widely used, it does not always reflect the degree of functional impairment of the nerve roots. Electrophysiological assessments, such as nerve conduction studies (NCS) and surface electromyography (sEMG), can provide additional information on neuromuscular function under conservative treatment. **Methods**: This quasi-experimental study included 60 patients with lumbar spondylosis and 25 healthy subjects, who underwent clinical, imaging, and electrophysiological assessments. NCS and sEMG parameters were assessed in the patient group before and six months after rehabilitation treatment. The control group was assessed only once, at baseline. We analyzed the nerve conduction velocity of the tibial and peroneal nerves and the sEMG activity of the tibialis anterior muscle bilaterally. Statistical analysis used nonparametric tests, Spearman’s coefficient, and Hodges–Lehmann estimates. **Results**: Compared to the control group, patients presented increased residual latencies and reduced CMAP amplitude and motor conduction velocity values (*p* < 0.001). After rehabilitation treatment, significant improvements in NCS parameters were observed, with decreased latencies and increased CMAP amplitude and motor conduction velocity bilaterally (*p* < 0.001). Also, sEMG amplitude and recruitment pattern scores increased significantly at the 6-month follow-up (*p* ≤ 0.004). Correlations between electrophysiological parameters and the severity of imaging changes were limited, with modest associations for left tibial latencies (ρ = 0.401–0.467; *p* < 0.050). **Conclusions**: In patients with lumbar spondylosis, rehabilitation treatment was associated with functional improvements in nerve conduction velocity parameters and muscle activity.

## 1. Introduction

Lumbar spondylosis is characterized by progressive degeneration of the spine, causing chronic back pain and limitations in physical activity in the adult population. Worldwide, low back pain has become a major cause of disability, affecting many people and having a significant impact on quality of life and work capacity [1]. Recent studies show that the impact of low back pain increases with age and is closely related to degenerative changes in the lumbar spine [1,2]. From a pathophysiological point of view, lumbar spondylosis is described as a slow degenerative process affecting the intervertebral disc, vertebral endplates, and facet joints. Intervertebral disc degeneration is characterized by a progressive reduction in fluid and proteoglycan content, alterations in the extracellular matrix’s structure, and modifications to biomechanical properties. These changes lead to an abnormal distribution of mechanical loads, affecting adjacent structural alterations [3]. Therefore, these processes can facilitate the development of disc protrusions, disc herniations, and related degenerative changes, which are commonly observed in lumbar spondylosis. Magnetic resonance imaging (MRI) is an important method for assessing degenerative changes to neural structures. In addition to this, numerous studies have shown that all imaging signs of spinal degeneration, including disc protrusions, are frequently seen in asymptomatic individuals, and the prevalence of these changes is increasing over time [4]. The findings also highlight the fact that structural changes in MRI are not directly proportional to symptomatology; attention should be paid to imaging data in order to evaluate clinical and functional assessment.

Patients with lumbar spondylosis can present varied symptoms, from mechanical low back pain to related neurological issues, especially when there are herniated discs or lumbar canal stenosis. Lumbar stenosis is a clinical syndrome characterized by a narrowing of the spinal canal or surrounding structures. However, the severity of these anatomical changes does not always match the severity of the symptoms or the degree of functional impairment [5]. As a result, the initial treatment usually involves conservative methods, with surgery reserved for specific cases. In the absence of indicators of progression, European clinical guidelines suggest conservative treatment as the initial course of treatment for patients with lumbar degenerative pathology and chronic low back pain [6]. In order to minimize pain, enhance function, and maximize neuromuscular control, medical rehabilitation and physical therapy are essential parts of this strategy. Although the results are impacted by the heterogeneity of the populations and protocols used, available systematic reviews support the idea that therapeutic exercise and rehabilitation interventions may lead to pain and disability relief in chronic low back pain [7].

Electrodiagnostic investigations can be used to enhance the functional assessment of patients with lumbar spondylosis. Nerve conduction studies (NCS) allow the analysis of latencies, amplitudes, and motor conduction velocities, which are useful in identifying and characterizing peripheral nerve damage and in differentiating it from other neurological entities [8,9]. Surface electromyography (sEMG) offers a non-invasive method of assessing muscle activity, allowing for the analysis of signal amplitude and muscle recruitment patterns, parameters related to neuromuscular control, and functional adaptations associated with low back pain and dysfunction [8].

Previous studies indicate that exercise-based interventions may influence muscle activation patterns and neuromuscular control in patients with non-specific low back pain, as reflected by changes in electrophysiological measures. Systematic reviews report significant effects of exercise interventions on EMG measures of lumbar muscle activity, supporting the sensitivity of electrophysiological parameters to functional adaptations after rehabilitation treatment [10]. Based on these findings, this study aimed to evaluate changes in NCS and sEMG parameters in patients with lumbar spondylosis who underwent rehabilitation treatment, compared with a healthy control group, over a six-month follow-up period. We hypothesized that patients with lumbar spondylosis would present altered electrophysiological parameters compared with healthy controls and that these parameters would improve following rehabilitation at the six-month follow-up.

## 2. Materials and Methods

### 2.1. Study Design

We conducted our investigation in a medical rehabilitation clinic in Timis County and included 60 patients diagnosed with lumbar spondylosis who underwent rehabilitation treatment between January 2023 and July 2025, along with 25 healthy subjects in a control group. The patient group was based on feasibility and patient availability during the study period. This quasi-experimental study aims to evaluate neurofunctional electrophysiological changes in patients diagnosed with lumbar spondylosis, as well as their association with clinical and imaging parameters. In this study, we assess neurofunctional electrophysiological parameters in patient group before rehabilitation treatment and after 6 months [11]. The study was conducted in accordance with the Declaration of Helsinki and approved by the Ethics Committee of Victor Babes University of Medicine and Pharmacy Timisoara, Romania (protocol no. 28/10.01.2022).

*Patient group*

The sample included 60 patients diagnosed with lumbar spondylosis, who were clinically, imaging, and electrophysiologically assessed. The diagnosis was established based on clinical examination and imaging investigations. Lumbar MRI findings included intervertebral disc degeneration, endplate Modic changes, facet arthropathy, and spinal canal or foraminal narrowing, in accordance with European guidelines for the assessment of degenerative lumbar spine disease [6]. Consecutive eligible patients attending the rehabilitation clinic during the study period were invited to participate. Inclusion criteria: age ≥ 18 years, confirmed diagnosis of lumbar spondylosis, presence of symptoms (low back pain ± radiculopathy), willingness to undergo initial electrophysiological evaluation and reevaluation after 6 months, and signed informed consent. Exclusion criteria: peripheral neuropathies of other etiologies, such as diabetic, alcoholic, toxic [12], history of spinal cord trauma, previous lumbar surgery, primary neuromuscular diseases, and severe systemic diseases with a potential impact on nerve conduction. Of the 60 patients included, not all had complete electrophysiological data for each nerve and muscle examined, due to technical or clinical reasons. However, the same nerves and muscles assessed at baseline were re-evaluated at the six-month follow-up for each participant to ensure within-subject comparability.

*Control group*

The control group included 25 healthy volunteers from the general population with no history of chronic low back pain, radiculopathy, or known neurological disorders. They were evaluated electrophysiologically once to obtain reference values for the investigated parameters. The control group was not matched to patients; potential differences in age, sex, or body composition between groups are acknowledged as possible confounding factors. The sample size may also limit statistical power.

*Clinical evaluation*

Clinical evaluation included: comprehensive anamnesis indicating low back pain duration (expressed in months); pain intensity evaluation using the visual analog scale (VAS), a validated method for low back pain assessment [13]; clinical response to rehabilitation treatment expressed as ΔVAS meaning difference between baseline (T0) and 6-month follow-up (T1) VAS scores; and the presence or absence of radiculopathy, defined as back pain radiating to the lower limb [14]. The radiculopathy variable was binary coded (0 = absent, 1 = present). No additional functional assessments were performed, which is acknowledged as a study limitation.

*Imaging evaluation*

In the patient group, 37 patients had lumbar MRI scans that showed degenerative disc changes associated with structural alterations. Following MRI examination, typical structural lesions were identified, namely disc protrusions, disc herniations, and spinal stenosis [4]. For statistical analysis, these were coded ordinally as follows: 1 = disc protrusions, 2 = disc herniation, and 3 = spinal stenosis. The remaining patients in the study group were evaluated using lumbar radiography, which revealed characteristic radiographic signs such as narrowing of the intervertebral spaces and osteophytosis. The radiographic investigations served to confirm the diagnosis and were not included in the clinical-imaging association analyses. All subjects in the control group had normal MRI scans, with no degenerative structural changes; therefore, the clinical-imaging association analyses were performed exclusively in the patient group.

*Electrophysiological assessment*

**Figure 1:**
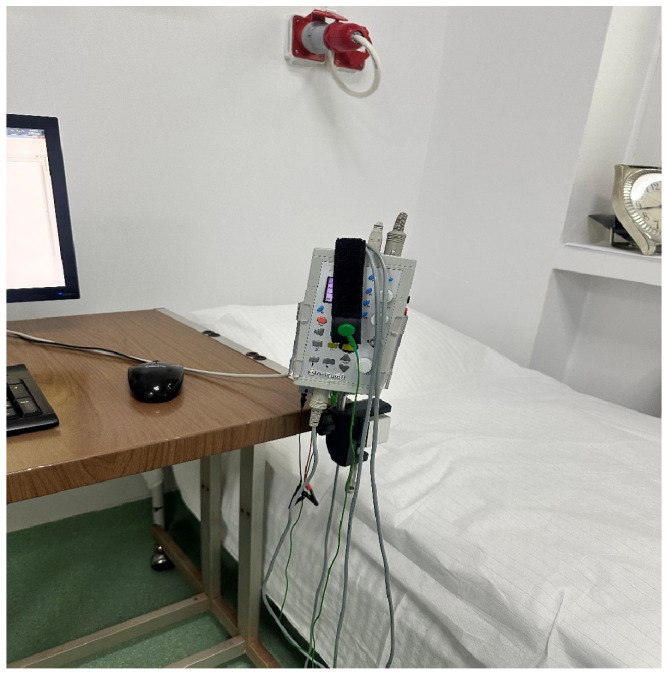
Neuro-MEP Micro device (2009 version; Neurosoft Ltd., Ivanovo, Russia).

**Figure 2:**
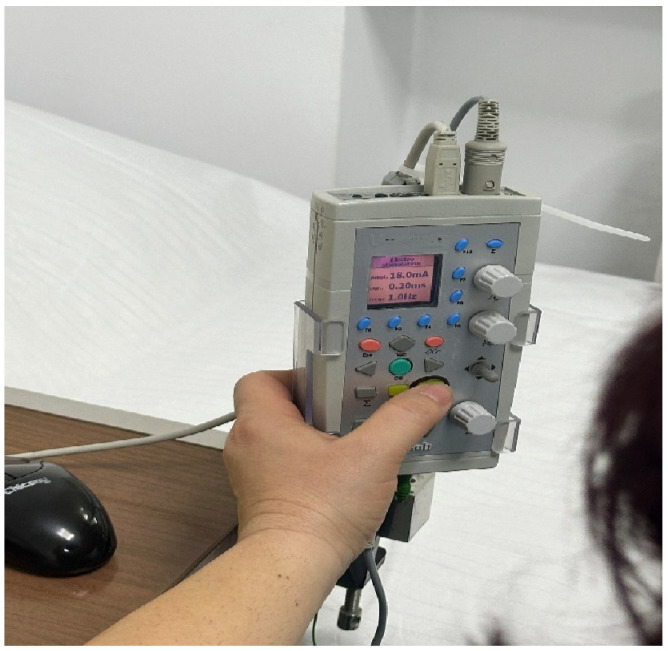
Control panel of the Neuro-MEP Micro device (2009 version; Neurosoft Ltd., Ivanovo, Russia).

Neurofunctional electrophysiological examinations were performed using the Neuro-MEP Micro device (2009 version; Neurosoft Ltd., Ivanovo, Russia) (Figure 1 and Figure 2) in a controlled environment with a minimum ambient temperature of 22 °C, in accordance with standard recommendations for electrodiagnostic investigations respecting stimulation techniques [15,16]. Limb skin temperature was not systematically monitored during nerve conduction studies, which represents a methodological limitation. In this study, the tibial and peroneal nerves were evaluated bilaterally.

*Examination procedure of nerve conduction studies*

**Figure 3:**
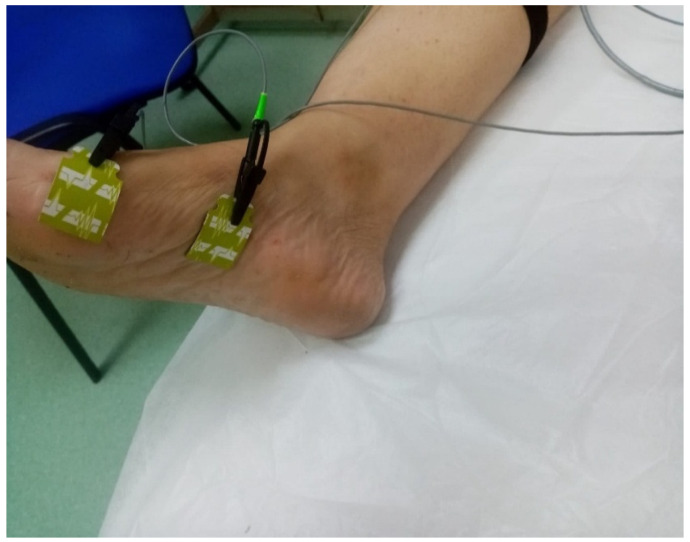
Electrodes placement for the NCS right tibial nerve assessment.

The patient was positioned in supine position with the lower limbs relaxed, while the skin was cleaned to reduce cutaneous impedance. Figure 3 illustrates the positioning of the electrodes for the assessment of the tibial nerve, recorded at the level of the abductor hallucis muscle. The active electrode was placed on the medial plantar surface of the foot, the distal reference electrode at the level of the forefoot, and the ground electrode on the calf.

**Figure 4:**
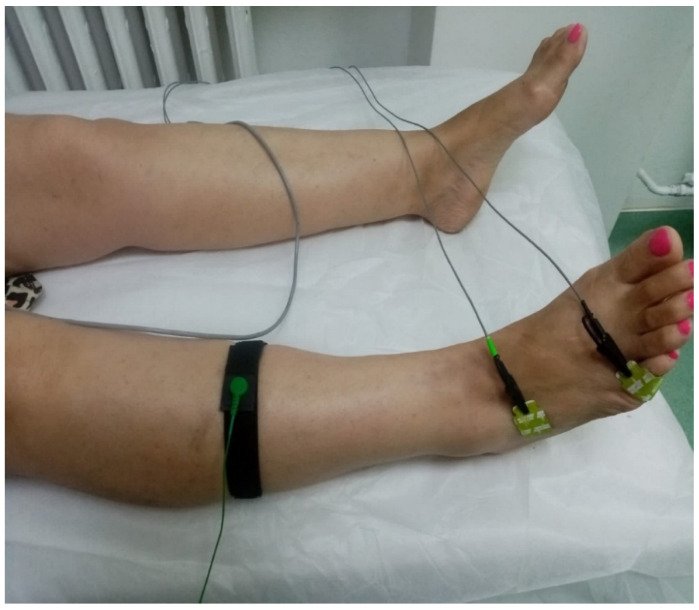
Electrode placement for the NCS right peroneal nerve assessment.

Electrical stimulation was performed percutaneously, at the ankle for the peroneal nerve (Figure 4) and at the medial malleolus for the tibial nerve. The intensity of the stimulus was gradually increased until a stable supramaximal response was obtained. The parameters analyzed for each nerve were as follows: distal motor latency (ms); residual latency; compound muscle action potential (CMAP) amplitude (mV); and motor nerve conduction velocity (m/s) [15,16,17]. The values obtained were compared with the reference values reported in the literature for motor NCS [17,18].

All measurements were performed bilaterally, and the values obtained were analyzed separately for each nerve examined. Electrophysiological assessments were repeated 6 months after the rehabilitation treatment program was applied to the patient group to analyze longitudinal changes. The control group underwent a single assessment, the initial one, which was used to obtain reference values. All examinations were performed by the same examiner to reduce observer variability. The examiner was not blinded to clinical or imaging data.

*Surface electromyography (sEMG)*

Electromyographic activity was assessed using the Neuro-MEP Micro device (2009 version). sEMG is a non-invasive method that allows for a global analysis of muscle activation using skin electrodes [8,19,20]. The tibialis anterior muscle was selected due to its predominant L4–L5 innervation and frequent involvement in lumbar radiculopathy [17].

*Examination procedure of sEMG*

**Figure 5:**
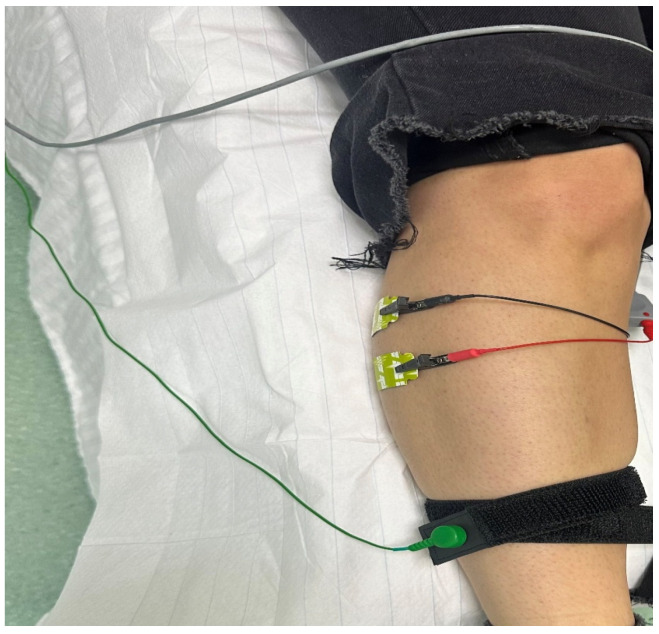
Electrodes placement for sEMG tibialis muscle.

Subjects were positioned in a supine position, relaxed, and skin in the area where the electrodes were applied was cleaned to reduce skin impedance [21]. The sEMG recordings in this study were performed using surface electrodes placed on the anterolateral side of the calf, longitudinally on the muscle fiber (Figure 5), according to SENIAM (Surface EMG for Non-Invasive Assessment of Muscles) recommendations [8].

After the electrodes were positioned, the subjects were asked to perform voluntary dorsiflexion contractions of the ankle, at the verbal command of the examiner, avoiding compensatory movements. The electromyographic activity of the tibialis anterior muscle was assessed using quantitative sEMG. This included the following parameters: sEMG signal amplitude, duration, and muscle recruitment pattern [8,19,20]. All sEMG assessments were performed bilaterally. For statistical analysis, electromyographic results were coded on an ordinal numerical scale (0–5) applied to each parameter analyzed, where a score of 5 indicated normal activity and a score of 0 indicated the absence of detectable electromyographic activity. Electrophysiological assessments were repeated 6 months after the rehabilitation treatment program was applied to the patient group, in order to analyze longitudinal changes. The control group underwent a single assessment, the initial one, which was used to obtain reference values. All neurophysiological assessments followed the same examination protocol and were performed by the same examiner to reduce observer variability. The examiner was not blinded to clinical or imaging data.

*Rehabilitation treatment program*

The first procedure used was balneotherapy, involving the application of CO_2_ mineral water baths at a temperature of 33 °C for 20 min daily. Baths were applied in rectangular acrylic tubs to ensure a consistent water temperature during the procedure. Once they finished this procedure, the study participants had one hour of rest, in accordance with the study protocol [22,23]. Following the rest period, we proceeded with an electrotherapy session, involving the application of interferential current (IFC), which is a medium-frequency electrical therapy, with four electrodes placed on the skin in a crossover position in the lumbar region; the intensity felt by patients was deep vibration. The frequency applied was 80–100 Hz to reach the analgesic threshold, and the duration was 20 min, in accordance with a recent study [24]. After the IFC therapy, we applied continuous ultrasound therapy at a frequency of 1 MHz and an intensity of 0.8 W/cm^2^ to the lumbar region, prior to the kinetotherapy exercises. The duration of application was adjusted to the treated area, according to the formula 1 min/5 cm^2^. The intensity chosen was within the range recommended in a recent study for chronic and degenerative lumbar conditions [25]. A thermotherapy session followed, consisting of rectangular paraffin wax sheets, 1–2 cm thick, placed on the lumbar area at a temperature of 40–45 degrees for 20 min. After this procedure, they had one hour of rest.

Kinetotherapy program was applied daily for 14 consecutive days to patients with lumbar spondylosis with and without radiculopathy and consisted of a standardized multimodal program. Each session included: lumbar and hip mobility exercises (pelvic tilts, cat–camel, hamstring and hip flexor stretching: 2 sets × 8–12 repetitions); lumbopelvic stabilization exercises (transverse abdominal activation 2 × 8–10 repetitions with 8–10 sec hold; McGill curl-up exercises, bilateral side bridge, bird-dog–10 repetitions/exercise; bridge 2 × 10 repetitions); and functional exercises (sit-to-stand and hip hinge: 2 × 8–10 repetitions). For patients with radiculopathy, the exercises were performed exclusively within limits that did not induce peripheralization of symptoms, adding neural mobilization (2 × 10–15 repetitions). This protocol is aligned with current recommendations for chronic and degenerative lumbar pain [26]. All interventions were applied in the same order for all subjects. The rehabilitation protocol was conducted in the clinic under supervision and applied identically to all patients (Table 1).

### 2.2. Statistical Analysis

Statistical analysis was performed using MedCalc software version 23.2.1 (MedCalc Software Ltd., Ostend, Belgium). In our study, we used the Shapiro–Wilk test to assess the distribution of continuous variables. Demographic and anthropometric variables were expressed as mean ± standard deviation, while clinical and electrophysiological variables were expressed as median and interquartile range [IQR]. The Mann–Whitney U test was used to compare continuous variables between independent groups, and the Wilcoxon test was used for paired samples. Hodges–Lehmann estimates with 95% confidence intervals were calculated to quantify effect size in nonparametric analyses; Spearman’s correlation coefficient was used to assess the association between clinical variables and electrophysiological parameters before and after rehabilitation treatment. A significance value of *p* < 0.05 was considered statistically significant. Multivariable linear regression analyses were performed to adjust for potential confounding factors, including age, sex, BMI, diabetes mellitus, and hypertension. They were used for both comparisons between patients and controls and for longitudinal analyses within the patient group. Regression coefficients (B) with 95% confidence intervals were reported. Based on the number of predictors included in the regression analysis, for a medium effect size and a power of 80%, at least 55 patients should be included in the study group (G*Power 3).

## 3. Results

### 3.1. Study Population Characteristics

The study included 85 subjects, with 60 patients diagnosed with lumbar spondylosis and 25 control subjects without a history of lumbar spondylosis. The patients’ ages ranged from 34 to 80 years. Compared with controls, patients were significantly older (*p* = 0.001) and had a higher body mass index (*p* = 0.009). Gender distribution did not differ significantly between groups, with women representing 61.7% of patients and 56.0% of controls (*p* = 0.629). The patients’ characteristics are presented in Table 2.

Comorbid conditions were present in both groups. However, arterial hypertension was significantly more prevalent in patients than in controls (65.0% vs. 28.0%, *p* = 0.001), as was uncomplicated diabetes mellitus (30.0% vs. 4.0%, *p* = 0.009). These baseline differences in age, BMI, and comorbidities were considered potential confounders for electrophysiological measurements and were included as covariates in subsequent multivariable analyses.

### 3.2. Clinical and Imaging Associations

Approximately 70% of patients reported prior use of nonsteroidal anti-inflammatory drugs, and 43% reported the use of weak opioid analgesics. The use of analgesic medication was associated with a significantly longer duration of low back pain (*p* < 0.001), whereas no significant association was observed between nonsteroidal anti-inflammatory drugs use and low back pain duration (*p* = 0.993). When examining imaging correlates, no significant differences in low back pain duration were observed across MRI-defined subgroups (disc protrusion, disc herniation, or spinal stenosis; Kruskal–Wallis test, *p* = 0.560). In contrast, from a clinical perspective, patients with radiculopathy had a significantly longer duration of low back pain compared with those without radiculopathy (Mann–Whitney U test, *p* < 0.001).

### 3.3. Pain Outcomes

Pain intensity was assessed using the 10-point visual analog scale (VAS). Median VAS scores decreased significantly from 5 [IQR 4–7] at baseline to 3 [IQR 2–4] at 6-month follow-up (*p* < 0.001), corresponding to a Hodges–Lehmann median reduction of 2 points (95% CI −2.5 to −1.5). The median change in VAS score (ΔVAS) was significantly greater in patients with radiculopathy compared with those without radiculopathy (*p* < 0.001). No significant correlation was observed between ΔVAS and duration of low back pain (Spearman’s ρ = 0.168, *p* = 0.201).

### 3.4. Comparisons of NCV Parameters Before Rehabilitation Treatment in Patients Group vs. Control Group

In our study, we observed at baseline that patients had significant abnormalities in NCS for the tibial nerve compared with controls. For the left tibial nerve, residual latency was significantly increased, while CMAP amplitude and motor conduction velocity (MCV) were significantly reduced (all *p* ≤ 0.001), whereas distal latency did not differ significantly between groups. The Hodges–Lehmann effect size estimation indicated clinically relevant between-group differences for residual latency, CMAP amplitude, and MCV (Table 3).

NCV parameters of the peroneal nerve also differed significantly between groups (Table 4). Patients showed significantly higher residual latency, lower CMAP amplitude, and reduced MCV values compared with controls (*p* ≤ 0.009), while distal latency did not differ significantly between groups (*p* > 0.155). The consistent preservation of distal latency, despite increased residual latency and reduced amplitude and MCV values in both nerves, suggests a neurophysiological pattern consistent with proximal nerve involvement, similar to findings reported in lumbar radiculopathy [27].

Multivariable linear regression analysis adjusted for age, sex, body mass index, diabetes mellitus, and hypertension confirmed persistent between-group differences for several NCV parameters of both tibial and peroneal nerves.

For the tibial nerve, patients had significantly prolonged distal latency, increased residual latency, reduced CMAP amplitude, and MCV on the right side compared with controls. On the left side, residual latency, CMAP amplitude, and MCV remained altered, whereas distal latency did not differ significantly between groups (Table 5). For the peroneal nerve, distal latency was not different between groups on either side (Table 6). However, patients showed increased residual latency and reduced CMAP amplitude bilaterally, while MCV was significantly decreased on both sides.

Our results indicate baseline neurophysiological impairment in patients compared with controls that persists after adjustment for major demographic and clinical covariates.

### 3.5. Assessment of Linear Regression Assumptions

Assumptions of the linear regression models were evaluated for NCV parameters. For the tibial nerve, deviations from residual normality were found for residual latency bilaterally, which was more pronounced on the right side. A similar result was seen in right distal latency, while the left side showed a mild deviation. Among peroneal nerve models, the most evident deviation was observed in left distal latency. For the longitudinal regression models, assumptions were examined for parameters in which the Shapiro–Wilk test indicated abnormal residuals, but we did not identify major issues.

The assumptions for sEMG parameters were evaluated in both the cross-sectional and longitudinal analyses. In cases that included recruitment pattern variables, the test indicated deviations from residual normality, which was expected given the ordinal character.

However, in all cases, visual inspection of residual vs. fitted plots did not suggest heteroscedasticity or nonlinearity, and multicollinearity remained within acceptable limits with a maximum VIF of 4.17. We did not identify structural problems, and all models were considered adequate for interpretation.

### 3.6. Longitudinal Evolution of NCV Parameters in Patients Group

In our study, significant abnormalities were observed at baseline (T0), and longitudinal analysis within the patient group demonstrated significant neurophysiological improvements at the 6-month follow-up. For the tibial nerve, both distal and residual latencies decreased, while CMAP amplitude and motor conduction velocity increased bilaterally. Hodges–Lehmann effect size estimates indicated relevant differences across both sides in longitudinal analysis (Table 7). A comparable pattern was observed for the peroneal nerve. At follow-up (T1), patients showed significant bilateral reductions in latency measures, together with increases in CMAP amplitude and MCV. The consistency of effect sizes across parameters and sides supports a symmetrical response to treatment (Table 8). After adjustment for covariates, most NCV parameters after rehabilitation treatment remained associated with their baseline values in both tibial and peroneal nerves. No significant association was observed for distal latency of the left peroneal nerve (Table 9).

### 3.7. Clinical–Electrophysiological Associations

In our study, Spearman correlation analysis showed limited associations between structural MRI findings and NCV parameters at baseline in the patient group. A modest correlation was observed between MRI severity in distal and residual latencies of the left tibial nerve (ρ = 0.467 and ρ = 0.401, respectively; *p* < 0.05), while no significant correlations were identified for motor conduction velocity or CMAP amplitude, nor for peroneal nerve parameters (Table 10).

A significant negative correlation was identified between symptom duration and CMAP amplitude of the right tibial and peroneal nerves (ρ = −0.328 and ρ = −0.300, respectively; *p* < 0.05), indicating lower motor amplitudes in patients with longer symptom duration. Additionally, symptom duration correlated positively with residual latency of the right peroneal nerve (ρ = 0.280, *p* = 0.044). No significant correlations were observed for motor conduction velocity (Table 11).

Patients with radiculopathy had significant abnormalities in motor nerve conduction parameters compared with patients without radiculopathy. For both the tibial and peroneal nerves, CMAP amplitudes were significantly reduced and residual latencies were significantly increased bilaterally (*p* < 0.010). Motor conduction velocity was also significantly lower in patients with radiculopathy for both nerves on both sides (*p* ≤ 0.019). In contrast, distal latency did not show consistent differences between groups, reaching statistical significance only for the right tibial nerve (*p* = 0.006), while remaining preserved for the left tibial nerve (*p* = 0.513) and for both sides of the peroneal nerve (*p* = 0.708 and *p* = 0.180). Hodges–Lehmann effect size estimates indicated substantial intergroup differences for CMAP amplitude and residual latency, and moderate differences for motor conduction velocity, supporting the ability of motor NCS parameters to discriminate patients with clinical radiculopathy (Table 12 and Table 13).

### 3.8. sEMG Characteristics in Patients and Controls

Patients with lumbar spondylosis and healthy controls had differences in sEMG assessments of the tibialis anterior muscle. The sEMG recruitment pattern and global signal amplitude were evaluated using a semi-quantitative ordinal scale with a range of 0 to 5, where 5 indicates a normal recruitment pattern or amplitude, 4 nearly normal, 3 mildly reduced, 2 moderately reduced, 1 severely reduced, and 0 absence of organized motor unit activity. Using this scale, sEMG recruitment pattern scores were significantly reduced in patients on both sides, with median values of 2 [IQR 2–3] corresponding to a Hodges–Lehmann median difference of 3 points (95% CI 2–3, *p* < 0.001) bilaterally. Similarly, sEMG amplitude scores were reduced in patients, with median values of 2 [IQR 2–4] on the right side and 3 [IQR 2–4] on the left side. The corresponding Hodges–Lehmann median differences were 3 points for the right tibialis anterior muscle (95% CI 1–3, *p* < 0.001) and 2 points for the left side (95% CI 1–3, *p* < 0.001). No significant differences were observed in the temporal features of the sEMG signal between groups. Multivariable analysis confirmed significantly lower recruitment pattern and sEMG amplitude scores in patients bilaterally compared with controls (all *p* < 0.001) after adjustment for covariables (Table 14).

### 3.9. sEMG Findings After Rehabilitation Treatment

At the six-month follow-up, sEMG parameters of the tibialis anterior muscle showed significant improvement compared with scores before rehabilitation treatment. Recruitment pattern scores increased bilaterally, from a median of 2 [IQR 2–3] at baseline to 3 [IQR 3–4] at follow-up, with Hodges–Lehmann median paired differences of 1.0 point on the right side and 0.5 points on the left side (both *p* < 0.001). Similarly, sEMG amplitude scores improved significantly, increasing from 2 [IQR 2–4] to 3.5 [IQR 3–4] on the right side (*p* = 0.001) and from 3 [IQR 2–4] to 3.5 [IQR 2.5–4] on the left side (*p* = 0.004), with Hodges–Lehmann median paired differences of 0.5 points bilaterally (Table 15). Multivariable linear regression analyses adjusting for baseline values showed that after rehabilitation treatment, sEMG parameters scores remained significantly associated with their corresponding baseline measurements on both sides (all *p* ≤ 0.011) (Table 16).

### 3.10. EMG–NCS Associations

In our study, before rehabilitation treatment, a significant negative correlation was observed between symptom duration and sEMG recruitment pattern of the left TA muscle (Spearman’s ρ = −0.369, *p* = 0.019), indicating lower recruitment scores in patients with longer symptom duration. In contrast, no significant association was identified on the right side (Spearman’s ρ = 0.226, *p* = 0.161), suggesting a side-specific relationship between symptom chronicity and neuromuscular impairment at presentation. Subsequent correlation analyses between symptom duration and changes in sEMG parameters following rehabilitation revealed no significant associations. Neither changes in sEMG amplitude nor recruitment pattern were significantly correlated on either side (all *p* ≥ 0.147), indicating that sEMG improvements after rehabilitation occurred independently of symptom duration.

Patients with clinical radiculopathy showed significantly a reduced sEMG recruitment pattern and amplitude scores of the TA muscle compared with those without radiculopathy (Table 17). These differences were observed bilaterally for both parameters, with Hodges–Lehmann median differences of 2.0 points for recruitment pattern and 1.0 points for amplitude on both sides (all *p* < 0.001). Overall, these findings indicate more pronounced neuromuscular impairment in patients with radiculopathy and may support the potential role of sEMG parameters as objective markers of neurological involvement.

Correlation analysis between sEMG and NCS parameters showed no significant associations between amplitude or recruitment pattern scores and CMAP amplitudes or residual latencies (*p* > 0.05). A modest positive correlation was observed between changes in sEMG amplitude and recruitment pattern following rehabilitation treatment for the left TA muscle (Spearman’s ρ = 0.329, *p* = 0.038), whereas no significant association was identified on the right side (Spearman’s ρ = 0.247, *p* = 0.125). Our results suggest a limited overlap between sEMG and NCV parameters, indicating that sEMG may capture aspects of neuromuscular function not fully reflected by nerve conduction measures.

## 4. Discussion

This study complements the neurofunctional assessment by including surface electromyography and assessing longitudinal changes over a six-month follow-up after rehabilitation treatment, based on our earlier observational report showing that NCS can detect functional abnormalities in lumbar radiculopathy even in the absence of clinical neurological deficit [28].

In our study, the assessment of patients with lumbar spondylosis demonstrated significant improvements in motor nerve conduction parameters after rehabilitation, characterized by decreases in distal and residual latencies and increases in CMAP amplitude and motor conduction velocity for the tibial and peroneal nerves, bilaterally (all *p* < 0.001). These results may suggest a progressive improvement in peripheral nerve function over time in the context of applying rehabilitation treatment.

Our data are consistent with studies that have evaluated the effects of rehabilitation treatment on lumbar radiculopathy. A recent study by Plaza-Manzano et al. showed that including neurodynamic mobilization in a motor control exercise program leads to a more pronounced improvement in neuropathic symptoms in patients with lumbar radiculopathy, and Karagül et al. reported a reduction in radicular pain after applying TENS and laser therapy, although the medium-term effects were more evident for lower limb pain [29,30]. Systematic reviews by Kuligowski et al. and Gameeva et al. support the effectiveness of manual and multimodal therapy interventions in reducing pain and improving function in patients with cervical and lumbar radiculopathy, including at 3–6-month follow-ups [31,32]. In this context, the clinical improvements observed after rehabilitation treatment accompanied by measurable electrophysiological changes may suggest a functional recovery of nerve conduction.

In another study, similar results were reported by Toyokura et al., who observed improvements in electrophysiological parameters after treatment, partially correlated with clinical recovery. This was also observed by Savage et al., who evidenced electrodiagnostic changes associated with favorable evolution under conservative treatment in sciatica [33,34].

Our results showed significant differences in NCV parameters before rehabilitation treatment, characterized by reduced CMAP amplitude and motor conduction velocity, as well as increased residual latency (*p* < 0.001). In addition, within the patient group, those with radiculopathy had significantly higher residual latency values and lower CMAP amplitudes compared to those without radiculopathy (*p* < 0.001 bilaterally), supporting the use of NCV parameters as functional markers of radicular severity, independent of structural changes detected by imaging. Our results showed a small correlation between electrophysiological parameters and the severity of MRI findings. This observation is supported by recent data in the literature; Montaner-Cuello et al. demonstrated limited concordance between MRI and electrodiagnostics in the evaluation of clinical suspicion of radiculopathy, and Rustom et al. did not identify significant associations between the severity of central or foraminal stenosis and electrodiagnostic confirmation [35,36]. These results suggest that MRI and electrodiagnostic assess different dimensions of radicular pathology: one structural, the other functional.

In our study, the assessment of sEMG parameters showed significant reductions in amplitude and recruitment pattern scores in patients compared to the control group (*p* < 0.001), suggesting decreased volitional muscle activation. Although both NCV and sEMG parameters improved significantly after rehabilitation treatment (*p* ≤ 0.004), they had small correlations, which may indicate that they capture different but complementary aspects of neuromuscular function. Overall, our results extend existing data on medical rehabilitation in patients with lumbar spondylosis, suggesting that conservative treatment may be associated with clinical improvements and electrophysiological changes. The improvements observed may be related to the multimodal rehabilitation program used in this study, which combined balneotherapy, thermotherapy, electrotherapy, and therapeutic exercise.

Our study has several limitations. The sample size was moderate, and no formal sample size calculation was performed, which may limit statistical power and the generalizability of the results. The absence of a randomized controlled group prevents a definitive attribution of the observed longitudinal changes after rehabilitation treatment. Electrophysiological assessment was limited to standard NCV studies and surface EMG; additional techniques, such as needle EMG, H-reflex, or F-wave analysis, could have provided complementary information. Imaging assessment was heterogeneous, including both MRI and radiography, which may not fully reflect functional nerve root impairment. Limb skin temperature was not systematically monitored during electrophysiological assessments, although examinations were performed in a temperature-controlled room. The study was not registered on a trial registry. Although multivariable analyses were used to adjust for potential confounding factors, the possibility of residual confounding cannot be ruled out. Follow-up was limited to six months, and functional or quality-of-life outcomes were not assessed. The examiner was not blinded to group allocation or clinical/imaging data, which should be considered when interpreting the results.

Despite these limitations, the results indicate that rehabilitation treatment was associated with improvements in NCV and sEMG parameters and reductions in pain intensity.

## 5. Conclusions

Longitudinal assessment in patients with lumbar spondylosis revealed significant improvements in NCS and sEMG parameters after rehabilitation treatment. These results support the complementary role of combining NCS and sEMG assessment in the functional evaluation and monitoring of patients undergoing rehabilitation treatment.

## Figures and Tables

**Table 1:** The rehabilitation treatment protocol in patient group.

Component	Description	Duration/Dosage	Frequency
Balneotherapy	CO_2_ mineral water bath, 33 °C	20 min/session	Daily, 14 days
IFC	Crossover lumbar application, frequency 80–100 Hz	20 min/session	Daily, 14 days
Ultrasound therapy	Paravertebral lumbar region, 0.8 W/cm^2^	1 min/5 cm^2^/session	Daily, 14 days
Thermotherapy	Paraffin wax sheets, 40–45 °C	20 min/session	Daily, 14 days
Kinetotherapy	Lumbar mobility exercises, core stabilization, stretching ± neural mobilization	40 min	Daily, 14 days

**Table 2:** Patients’ characteristics.

	Patients (*n* = 60)	Control group (*n* = 25)
Age (years) mean ± SD	55.5 ± 12.85	45.2 ± 13.2
Weight (kg) mean ± SD	75.31 ± 14.3	72.52 ± 17.1
Height (cm) mean ± SD	166.05 ± 9.28	170.16 ± 11.42
BMI (kg/m^2^)	27.19 ± 4.02	24.75 ± 3.7

**Table 3:** Comparison of NCV parameters at baseline (T0)—Right/Left Tibial Nerve (Patients vs. Controls).

Parameter	Patients T0 Right Tibial Nerve, Median [IQR]	Controls Right Tibial Nerve, Median [IQR]	Hodges–Lehmann (95% CI)	*p*-Value	Patients T0 Left Tibial Nerve, Median [IQR]	Controls Left Tibial Nerve, Median [IQR]	Hodges–Lehmann (95% CI)	*p*-Value
Distal latency (ms)	3.40 [3.03–3.80]	2.50 [2.08–3.50]	−0.90	0.003	3.55 [3.00–4.30]	3.50 [2.83–4.30]	−0.20	0.537
Residual latency (ms)	1.42 [1.20–2.05]	1.10 [0.86–1.31]	−0.45	0.001	1.58 [1.14–2.25]	1.08 [0.97–1.37]	−0.45	0.001
CMAP amplitude (mV)	8.70 [5.33–11.75]	13.10 [10.65–15.23]	4.30	<0.001	9.05 [5.90–11.70]	13.60 [12.23–15.05]	4.50	<0.001
Motor conduction velocity (m/s)	45.10 [41.03–48.20]	48.40 [47.88–49.10]	3.50	0.001	42.25 [40.20–47.20]	48.60 [47.60–49.10]	6.05	<0.001

Values are reported as median (interquartile range). Between-group comparisons were performed using the Mann–Whitney U test due to non-normal data distribution. Effect size was estimated using the Hodges–Lehmann estimator and is reported as the median difference between groups with 95% confidence intervals. Positive Hodges–Lehmann values indicate higher values in patients compared with controls, whereas negative values indicate lower values.

**Table 4:** Comparison of NCV parameters at baseline (T0)—Right/Left Peroneal Nerve (Patients vs. Controls).

Parameter	Patients T0 Right Peroneal Nerve, Median [IQR]	Controls Right Peroneal Nerve, Median [IQR]	Hodges–Lehmann (95% CI)	*p*-Value	Patients T0 Left Peroneal Nerve, Median [IQR]	Controls Left Peroneal Nerve, Median [IQR]	Hodges–Lehmann (95% CI)	*p*-Value
Distal latency (ms)	3.25 [2.85–3.80]	3.50 [2.50–4.13]	0.10	0.790	3.40 [2.90–4.00]	3.80 [2.90–4.48]	0.30	0.155
Residual latency (ms)	1.92 [1.66–2.36]	1.20 [0.90–1.42]	−0.79	<0.001	1.97 [1.55–2.42]	1.10 [0.91–1.48]	−0.85	<0.001
CMAP amplitude (mV)	4.25 [2.80–5.70]	12.60 [10.98–13.53]	8.20	<0.001	4.05 [2.80–6.80]	11.30 [10.20–12.45]	7.00	<0.001
Motor conduction velocity (m/s)	44.45 [41.20–46.90]	47.60 [46.27–48.70]	3.10	0.001	44.20 [41.40–48.00]	47.10 [46.18–48.00]	2.55	0.009

Data are presented as median and interquartile range (IQR). Between-group comparisons were performed using the Mann–Whitney U test. Effect size is reported as the Hodges–Lehmann estimator with 95% confidence intervals. A *p*-value < 0.05 was considered statistically significant.

**Table 5:** Multivariable linear regression analysis of baseline NCV parameters (patients vs. controls) for the tibial nerve.

Parameter	B Coefficient (Group) Right Side	95% Confidence Interval (Right Side)	*p*-Value	B Coefficient (Group) Left Side	95% Confidence Interval (Left Side)	*p*-Value
Distal latency (ms)	1.02	0.50 to 1.53	<0.001	0.21	−0.37 to 0.79	0.469
Residual latency (ms)	0.80	0.31 to 1.28	0.002	0.56	0.13 to 0.99	0.012
CMAP amplitude (mV)	−3.21	−5.19 to −1.23	0.002	−3.37	−5.64 to −1.10	0.004
Motor conduction velocity (m/s)	−3.84	−5.80 to −1.87	<0.001	−5.09	−7.14 to −3.03	<0.001

Multivariable linear regression analysis adjusting for age, sex, body mass index, diabetes mellitus, and hypertension. B coefficients represent the adjusted difference between patients and controls.

**Table 6:** Multivariable linear regression analysis of baseline NCV parameters (patients vs. controls) for the peroneal nerve.

Parameter	B Coefficient (Group) Right Side	95% Confidence Interval (Right Side)	*p*-Value	B Coefficient (Group) Left Side	95% Confidence Interval (Left Side)	*p*-Value
Distal latency (ms)	0.01	−0.56 to 0.59	0.969	0.14	−1.52 to 1.79	0.871
Residual latency (ms)	0.88	0.44 to 1.31	<0.001	0.86	0.57 to 1.15	<0.001
CMAP amplitude (mV)	−7.39	−8.47 to −6.30	<0.001	−6.53	−7.71 to −5.35	<0.001
Motor conduction velocity (m/s)	−4.19	−6.01 to −2.38	<0.001	−2.47	−4.58 to −0.37	0.022

Multivariable linear regression analysis adjusting for age, sex, body mass index, diabetes mellitus, and hypertension. B coefficients represent the adjusted difference between patients and controls.

**Table 7:** Results of NCV parameters in Right/left Tibial Nerve (T0 vs. T1, Patients group).

Parameter	T0 Right Tibial Nerve, Median [IQR]	T1 Right Tibial Nerve, Median [IQR]	Hodges–Lehmann (95% CI)	*p*-Value	T0 Left Tibial Nerve, Median [IQR]	T1 Left Tibial Nerve, Median [IQR]	Hodges–Lehmann (95% CI)	*p*-Value
Distal latency (ms)	3.4 [3.03–3.80]	3.0 [2.65–3.35]	−0.45	<0.001	3.55 [3.00–4.30]	3.00 [2.60–3.60]	−0.50	<0.001
Residual latency (ms)	1.42 [1.20–2.05]	1.20 [0.90–1.75]	−0.27	<0.001	1.58 [1.14–2.25]	1.35 [1.00–1.80]	−0.25	<0.001
CMAP amplitude (mV)	8.7 [5.33–11.75]	9.4 [6.95–13.45]	1.08	<0.001	9.05 [5.90–11.70]	10.00 [7.00–12.90]	1.10	0.001
Motor conduction velocity (m/s)	45.1 [41.03–48.20]	46.0 [43.00–49.00]	1.35	<0.001	42.25 [40.20–47.20]	44.00 [42.00–48.00]	1.55	<0.001

Values are reported as median (interquartile range). Paired pre–post comparisons were performed using the Wilcoxon signed-rank test. Effect size was estimated using the Hodges–Lehmann estimator, reported as the median of paired differences with 95% confidence intervals. Negative Hodges–Lehmann values indicate reductions in latency parameters, while positive values indicate increases in CMAP amplitude and motor conduction velocity.

**Table 8:** Results of NCV parameters in Right/Left Peroneal Nerve (T0 vs. T1, Patients group).

Parameter	T0 Right Peroneal Nerve, Median [IQR]	T1 Right Peroneal Nerve, Median [IQR]	Hodges–Lehmann (95% CI)	*p*-Value	T0 Left Peroneal Nerve, Median [IQR]	T1 Left Peroneal Nerve, Median [IQR]	Hodges–Lehmann (95% CI)	*p*-Value
Distal latency (ms)	3.25 [2.85–3.80]	2.80 [2.20–3.10]	−0.50	<0.001	3.40 [2.90–4.00]	2.80 [2.40–3.20]	−0.55	<0.001
Residual latency (ms)	1.92 [1.66–2.36]	1.60 [1.35–1.90]	−0.32	<0.001	1.97 [1.55–2.42]	1.69 [1.30–2.00]	−0.33	<0.001
CMAP amplitude (mV)	4.25 [2.80–5.70]	5.85 [4.95–7.00]	1.45	<0.001	4.05 [2.80–6.80]	5.15 [4.00–8.00]	1.40	<0.001
Motor conduction velocity (m/s)	44.45 [41.20–46.90]	45.80 [43.00–48.00]	1.15	<0.001	44.20 [41.40–48.00]	45.00 [43.00–48.90]	1.10	<0.001

Values are reported as median (interquartile range). Paired pre–post comparisons were performed using the Wilcoxon signed-rank test. Effect size was estimated using the Hodges–Lehmann estimator, reported as the median of paired differences with 95% confidence intervals. Negative Hodges–Lehmann values indicate reductions in latency parameters, while positive values indicate increases in CMAP amplitude and motor conduction velocity.

**Table 9:** Multivariable linear regression analysis of post-treatment NCV parameters (T1) adjusted for baseline values (T0) in the patient group.

Nerve	Parameter	Side	B Coefficient (T0-Baseline)	95% Confidence Interval	*p*-Value
Tibial	Distal latency (ms)	Right	0.84	0.69 to 0.98	<0.001
Tibial	Distal latency (ms)	Left	0.72	0.58 to 0.86	<0.001
Tibial	Residual latency (ms)	Right	0.75	0.68 to 0.82	<0.001
Tibial	Residual latency (ms)	Left	0.65	0.56 to 0.75	<0.001
Tibial	CMAP amplitude (mV)	Right	0.89	0.80 to 0.97	<0.001
Tibial	CMAP amplitude (mV)	Left	0.86	0.77 to 0.96	<0.001
Tibial	Motor conduction velocity (m/s)	Right	0.80	0.67 to 0.93	<0.001
Tibial	Motor conduction velocity (m/s)	Left	0.67	0.54 to 0.79	<0.001
Peroneal	Distal latency (ms)	Right	0.72	0.58 to 0.87	<0.001
Peroneal	Distal latency (ms)	Left	−0.00	−0.06 to 0.05	0.930
Peroneal	Residual latency (ms)	Right	0.67	0.61 to 0.74	<0.001
Peroneal	Residual latency (ms)	Left	0.68	0.56 to 0.80	<0.001
Peroneal	CMAP amplitude (mV)	Right	1.01	0.84 to 1.18	<0.001
Peroneal	CMAP amplitude (mV)	Left	1.06	0.90 to 1.22	<0.001
Peroneal	Motor conduction velocity (m/s)	Right	0.71	0.58 to 0.84	<0.001
Peroneal	Motor conduction velocity (m/s)	Left	0.83	0.73 to 0.92	<0.001

Multivariable linear regression analysis was performed with post-treatment values (T1) as dependent variables, adjusting for baseline values (T0), age, sex, BMI, diabetes mellitus, and hypertension. B coefficients represent the adjusted association between baseline and post-treatment values.

**Table 10:** Correlation analysis between structural MRI and functional NCV parameters on the patient group in the tibial (**A**) and peroneal nerve (**B**).

(A)								
	Right Tibial Nerve, Distal Latency (ms)	Left Tibial Nerve, Distal Latency (ms)	Right Tibial Nerve, Residual Latency (ms)	Left Tibial Nerve, Residual Latency (ms)	Right Tibial Nerve, CMAP Amplitude (mV)	Left Tibial Nerve, CMAP Amplitude (mV)	Right Tibial Nerve, MCV(m/s)	Left Tibial Nerve, MCV(m/s)
Spearman’s ρ	0.331	0.467	0.283	0.401	−0.051	−0.256	−0.275	−0.295
*p*-value	0.0920	0.0140	0.1520	0.0383	0.7985	0.1974	0.1657	0.1348
(**B**)								
	**Right Peroneal Nerve, Distal Latency (ms)**	**Left Peroneal Nerve, Distal Latency (ms)**	**Right Peroneal Nerve, Residual Latency (ms)**	**Left Peroneal Nerve, Residual Latency (ms)**	**Right Peroneal Nerve, CMAP Amplitude (mV)**	**Left Peroneal Nerve, CMAP Amplitude (mV)**	**Right Peroneal Nerve, MCV(m/s)**	**Left Peroneal Nerve, MCV(m/s)**
Spearman’s ρ	0.139	0.303	0.062	0.209	−0.201	−0.243	−0.276	−0.208
*p*-value	0.4654	0.1099	0.7437	0.2757	0.2872	0.2035	0.1391	0.2795

**Table 11:** Correlation analysis between symptom duration and functional NCV parameters on the patient group in the tibial (**A**) and peroneal nerve (**B**).

(A)								
	Right Tibial Nerve, Distal Latency (ms)	Left Tibial Nerve, Distal Latency (ms)	Right Tibial Nerve, Residual Latency (ms)	Left Tibial Nerve, Residual Latency (ms)	Right Tibial Nerve, CMAP Amplitude (mV)	Left Tibial Nerve, CMAP Amplitude (mV)	Right Tibial Nerve, MCV(m/s)	Left Tibial Nerve, MCV(m/s)
Spearman’s ρ	−0.0630	0.0735	−0.0129	−0.0132	−0.328	−0.188	−0.0209	−0.140
*p*-value	0.6880	0.6439	0.9347	0.9341	0.0318	0.2344	0.8942	0.3762
(**B**)								
	**Right Peroneal Nerve,** **Distal Latency (ms)**	**Left Peroneal Nerve,** **Distal Latency (ms)**	**Right Peroneal Nerve,** **Residual Latency (ms)**	**Left Peroneal Nerve,** **Residual Latency (ms)**	**Right Peroneal Nerve,** **CMAP Amplitude (mV)**	**Left Peroneal Nerve,** **CMAP Amplitude (mV)**	**Right Peroneal Nerve, MCV(m/s)**	**Left Peroneal Nerve, MCV(m/s)**
Spearman’s ρ	0.277	0.0281	0.280	0.216	−0.300	−0.144	−0.190	−0.207
*p*-value	0.0470	0.8459	0.0442	0.1321	0.0305	0.3174	0.1771	0.1501

**Table 12:** Hodges–Lehmann effect sizes for NCV parameters in the tibial nerve according to radiculopathy.

Effect Size	CMAP Amplitude (mV) Right Tibial Nerve	CMAP Amplitude (mV) Left Tibial Nerve	Residual Latency (ms) Right Tibial Nerve	Residual Latency (ms) Left Tibial Nerve	Distal Latency (ms), Right Tibial Nerve	Distal Latency (ms), Left Tibial Nerve	Motor Conduction Velocity (m/s), Right Tibial Nerve	Motor Conduction Velocity (m/s), Left Tibial Nerve
Hodges–Lehmann Median Difference	4.8	4.7	−0.34	−0.35	−0.60	−0.20	3.2	5.35
*p*-value	<0.001	<0.001	0.005	0.010	0.006	0.513	0.002	<0.001

**Table 13:** Hodges–Lehmann effect sizes for NCV parameters in the peroneal nerve according to radiculopathy.

Effect Size	CMAP Amplitude (mV), Right Peroneal Nerve	CMAP Amplitude (mV), Left Peroneal Nerve	Residual Latency (ms), Right Peroneal Nerve	Residual Latency (ms), Left Peroneal Nerve	Distal Latency (ms), Right Peroneal Nerve	Distal Latency (ms), Left Peroneal Nerve	Motor Conduction Velocity (m/s), Right Peroneal Nerve	Motor Conduction Velocity (m/s), Left Peroneal Nerve
Hodges–Lehmann Median Difference	7.2	5.85	−0.63	−0.63	−1.00	0.30	2.70	2.30
*p*-value	<0.001	<0.001	<0.001	<0.001	0.708	0.180	0.008	0.019

**Table 14:** Multivariable linear regression analysis of baseline sEMG parameters (patients vs. controls) for TA muscle.

Parameter	B Coefficient (Group) Right Side	95% Confidence Interval (Right Side)	*p*-Value	B Coefficient (Group) Left Side	95% Confidence Interval (Left Side)	*p*-Value
Recruitment pattern	−2.66	−2.92 to −2.40	<0.001	−2.43	−2.74 to −2.12	<0.001
Amplitude	−2.45	−2.87 to −2.02	<0.001	−1.96	−2.37 to −1.56	<0.001

Multivariable linear regression analysis adjusted for age, sex, body mass index, diabetes mellitus, and hypertension. Group coded as 1 = patients and 0 = controls. B coefficients represent the adjusted mean difference between patients and controls. Negative values indicate lower scores in patients. A *p*-value < 0.05 was considered statistically significant.

**Table 15:** sEMG parameters of the tibialis anterior muscle at T0 and T1 (patient group).

Parameter	Side	T0, Median [IQR]	T1, Median [IQR]	Hodges–Lehmann (95% CI)	*p*-Value
Recruitment pattern	Right	2 [2, 3]	3 [3, 4]	1.0 (0.5–1.0)	<0.001
Recruitment pattern	Left	2 [2, 3]	3 [3, 4]	0.5 (0.5–1.0)	<0.001
Amplitude	Right	2 [2–4]	3.5 [3, 4]	0.5 (0–1.0)	0.001
Amplitude	Left	3 [2–4]	3.5 [2.5–4]	0.5 (0–0.5)	0.004

Data are presented as median [IQR]. Paired comparisons were performed using the Wilcoxon signed-rank test. Hodges–Lehmann estimates represent median paired differences.

**Table 16:** Multivariable linear regression analysis of post-treatment sEMG parameters (T1) adjusted for baseline values (T0) in the patient group.

Parameter	Side	B Coefficient (T0-Baseline)	95% Confidence Interval	*p*-Value
Recruitment pattern	Right	0.53	0.13 to 0.92	0.011
Recruitment pattern	Left	0.80	0.51 to 1.09	<0.001
Amplitude	Right	0.41	0.14 to 0.69	0.004
Amplitude	Left	0.64	0.35 to 0.93	0.001

**Table 17:** Comparison of baseline sEMG recruitment pattern and amplitude according to the presence of radiculopathy.

Effect Size	sEMG Recruitment Pattern, Right Side	sEMG Recruitment Pattern, Left Side	sEMG Amplitude, Right Side	sEMG Amplitude, Left Side
Hodges–Lehmann Median Difference	2.00	2.00	1.00	1.00
*p*-value	<0.001	<0.001	<0.001	<0.001

## Data Availability

The data presented in this study are available upon request from the corresponding author (R.O.O.) and are not publicly available due to privacy restrictions.

## Abbreviations

The following abbreviations are used in this manuscript:

MRI	Magnetic resonance imaging
NCS	Nerve conduction studies
NCV	Nerve conduction velocity
EMG	Electromyography
sEMG	Surface electromyography
TA	Tibialis anterior
VAS	Visual analog scale
T0	Baseline assessments
T1	After rehabilitation assessments
CMAP	Compound muscle action potential
MCV	Motor conduction velocity
SENIAM	Surface EMG for Non-Invasive Assessment of Muscles
CO_2_	Carbon dioxide
IFC	Interferential current
BMI	Body mass index
HL	Hodges–Lehmann median difference
W/cm^2^	Watts per square centimeter
TENS	Transcutaneous electrical nerve stimulation
